# Editorial for the Special Issue “Foodborne and Waterborne Pathogens”

**DOI:** 10.3390/microorganisms14071554

**Published:** 2026-07-16

**Authors:** Yosra A. Helmy, Hafez M. Hafez

**Affiliations:** 1 Department of Veterinary Science, Martin-Gatton College of Agriculture, Food, and Environment, University of Kentucky, Lexington, KY 40546, USA; 2 Institute of Poultry Diseases, Freie Universität Berlin, 12309 Berlin, Germany

Foodborne and waterborne pathogens remain major global public health concerns, affecting human health, animal health, food safety, water quality, environmental health, and economic stability. Unsafe food contaminated with bacteria, viruses, parasites, or chemical substances can cause more than 200 diseases, ranging from mild gastrointestinal illness to severe systemic complications and cancer [1,2]. Globally, approximately one in ten people become ill each year after consuming contaminated food, resulting in an estimated 420,000 deaths annually [1]. Similarly, unsafe drinking water, inadequate sanitation, and poor hygiene continue to contribute substantially to diarrheal disease, with approximately one million deaths estimated each year due to unsafe drinking water, sanitation, and hand hygiene [3]. These burdens are disproportionately severe among young children, older adults, immunocompromised individuals, and populations in low- and middle-income countries with limited access to safe water, sanitation, food safety systems, and healthcare infrastructure.

The emergence and persistence of foodborne and waterborne pathogens are influenced by complex interactions among humans, animals, food production systems, water systems, agriculture, wildlife, environmental contamination, climate change, international travel, and global food trade. Pathogens such as *Salmonella*, *Campylobacter*, pathogenic *Escherichia coli*, *Listeria monocytogenes*, *Yersinia enterocolitica*, *Vibrio cholerae*, norovirus, hepatitis A virus, *Cryptosporidium*, and *Giardia* continue to pose substantial risks to public health [4,5,6,7,8]. In addition, antimicrobial resistance has become an increasingly urgent concern because resistant bacteria and antimicrobial resistance genes can disseminate through food, water, animals, humans, wastewater, and the environment [9]. Therefore, integrated One Health approaches are essential for understanding pathogen transmission, identifying reservoirs, improving genomic surveillance, developing novel diagnostics, and designing effective prevention and control strategies [10,11,12,13,14].

The Special Issue “Foodborne and Waterborne Pathogens” was established to provide a platform for research and review articles addressing the diagnosis, epidemiology, risk assessment, host–pathogen interactions, virulence, immune responses, antimicrobial resistance, environmental reservoirs, One Health perspectives, novel therapies, emerging zoonotic diseases, and approaches to reduce antibiotic use in food-producing animals. The five articles published in this Special Issue reflect the diversity of this field and highlight the need for interdisciplinary strategies to reduce the burden of foodborne and waterborne diseases.

Baráti-Deák et al. investigated the inhibitory activity of excreted metabolites produced by *Serratia marcescens* strains isolated from a dairy-producing environment against several important foodborne bacterial pathogens, including *Listeria monocytogenes*, *Salmonella hartford*, *Yersinia enterocolitica*, and *Escherichia coli* [15]. The study showed that a non-pigmented *S. marcescens* strain inhibited multiple pathogens, whereas a pigment-producing strain inhibited only *Y. enterocolitica*. The authors further demonstrated that concentrated cell-free supernatants had stronger inhibitory activity, supporting the extracellular nature of the antagonistic compounds. Although protease and chitinase enzymes appeared to contribute to this activity, they were not considered the primary inhibitory factors. The study suggested that combinations of extracellular metabolites may be responsible for the observed antimicrobial effects. These findings are important because they support the potential use of microbial-derived metabolites as bio-disinfectants or alternative antimicrobial tools in food-processing environments.

Chen and Meng addressed an important methodological challenge in food and water safety surveillance by evaluating short-read assemblers for metagenomic identification of foodborne and waterborne pathogens using simulated bacterial communities [16]. The study simulated bacterial communities from fresh spinach and surface water and included multidrug-resistant *Salmonella Indiana* and *Pseudomonas aeruginosa* as target organisms. Several assemblers, including ABySS, IDBA-UD, MaSuRCA, MEGAHIT, metaSPAdes, and Ray Meta, were benchmarked for assembly quality and downstream genomic analyses, including detection of plasmids, virulence genes, antimicrobial resistance genes, chromosomal point mutations, serotyping, multilocus sequence typing, and phylogenetic analysis. The authors found that MEGAHIT, metaSPAdes, and Ray Meta generally performed better for metagenomic identification, while higher sequencing depth improved accuracy. This work highlights the promise of metagenomics for pathogen detection in complex food and water microbiomes, while also emphasizing the need for continuous benchmarking and validation of bioinformatic workflows.

Sowah et al. examined the sources and drivers of antibiotic resistance genes in urban streams in Atlanta, Georgia, USA [17]. The study quantified sewage-associated markers, fecal indicator bacteria, the class 1 integron gene, and multiple antibiotic resistance genes, including *sulI*, *sulII*, *tetW*, *tetM*, *ampC*, and *blaSHV*, in stream water samples. The authors found that antibiotic resistance genes were widely distributed in the watershed and were strongly associated with sewage and fecal contamination markers. Their modeling analysis indicated that fecal source loading explained more than 70% of the variation in antibiotic resistance gene loads, while environmental factors and the class 1 integron gene contributed less. These findings are significant because they demonstrate that anthropogenic fecal pollution can be a major driver of antimicrobial resistance dissemination in aquatic environments. The study provides valuable information for watershed management and supports the inclusion of environmental water systems in One Health antimicrobial resistance surveillance.

Helmy and Hafez reviewed cryptosporidiosis as an important foodborne and waterborne zoonotic disease caused by protozoan parasites of the genus *Cryptosporidium* [18]. The review summarized key aspects of the parasite’s infective stage, life cycle, pathogenesis, genomics, epidemiology, outbreak history, transmission dynamics, host range, risk factors, diagnosis, treatment, prevention, and vaccine prospects. The authors emphasized that *C. hominis* and *C. parvum* are the main species associated with infections in humans and animals and that the resistant oocyst stage contributes to environmental persistence and transmission. The review also highlighted major control challenges, including resistance of oocysts to commonly used disinfectants such as chlorine and the lack of fully effective therapeutics or vaccines. By framing cryptosporidiosis within a One Health context, the article underscored the importance of integrated animal, human, and environmental approaches to reduce transmission and disease burden.

Wang provided a comprehensive review of mathematical models for cholera dynamics [19]. Cholera remains a major public health burden in many regions of the world, especially where access to safe drinking water, sanitation, and hygiene is limited. The review described classical cholera transmission models and discussed model extensions that incorporate spatial and temporal heterogeneity, environmental reservoirs, disease control strategies, human behavior, and multi-scale infection dynamics. The article emphasized that mathematical modeling is a valuable tool for understanding cholera transmission, predicting outbreaks, evaluating interventions, and guiding public health decision-making. Importantly, the review also identified challenges and opportunities for future modeling efforts, including the need for collaboration across modeling groups and disciplines. This contribution illustrates how computational and mathematical approaches can strengthen preparedness and response strategies for waterborne diseases.

Collectively, the articles in this Special Issue emphasize several important themes. First, pathogen detection and characterization are rapidly evolving through the integration of metagenomics, molecular diagnostics, and advanced bioinformatics. These tools can improve the detection of pathogens, antimicrobial resistance genes, virulence determinants, plasmids, and strain-level relationships directly from complex food and water samples. However, their successful application requires careful validation, standardization, and interpretation. Second, antimicrobial resistance is a critical challenge at the food–water–human–animal–environment interface. Environmental waters can act as reservoirs and transmission routes for resistant bacteria and resistance genes, particularly when impacted by sewage, agricultural runoff, urbanization, and other anthropogenic activities. Third, novel control strategies, including microbial metabolites and other non-antibiotic approaches, are needed to complement traditional interventions such as sanitation, hygiene, water treatment, food safety management, and antimicrobial stewardship.

This Special Issue also reinforces the importance of a One Health framework. Foodborne and waterborne pathogens do not exist within isolated systems. Their emergence, persistence, and transmission are shaped by interactions among microbial communities, hosts, food systems, water systems, environmental reservoirs, climate, human behavior, and public health infrastructure. Addressing these pathogens requires collaboration among microbiologists, veterinarians, physicians, epidemiologists, food safety specialists, environmental scientists, modelers, public health professionals, policymakers, and industry partners. Such interdisciplinary collaboration is essential for developing effective surveillance systems, identifying contamination sources, predicting disease risks, and implementing sustainable interventions.

In conclusion, the papers published in this Special Issue provide valuable insights into the biology, detection, ecology, transmission, resistance, and control of some foodborne and waterborne pathogens. They highlight important advances in antimicrobial alternatives, metagenomic surveillance, environmental antimicrobial resistance monitoring, parasitic disease control, and mathematical modeling of waterborne disease dynamics. At the same time, they reveal important knowledge gaps, including the need for improved surveillance across the food–water–animal–human interface, standardized genomic and metagenomic workflows, better understanding of environmental reservoirs, development of safe and effective non-antibiotic interventions, and stronger translation of research findings into public health practice. Continued interdisciplinary and One Health-oriented research will be essential to reduce the global burden of foodborne and waterborne diseases and to protect human, animal, and environmental health.
